# Antibiotic Prescription Patterns in Poland in the Years 2019–2024 Based on Reimbursement Data from the National Healthcare Fund

**DOI:** 10.3390/antibiotics15010015

**Published:** 2025-12-21

**Authors:** Aleksandra Danieluk, Sławomir Chlabicz

**Affiliations:** Department of Family Medicine, Medical University of Białystok, 15-054 Białystok, Poland

**Keywords:** antibiotic stewardship, point-of-care testing, antibiotic resistance

## Abstract

**Background/Objectives**: The global response to the COVID-19 pandemic included a notable shift in antibiotic prescribing patterns, with use declining and then rising again as restrictions were lifted. In Poland, point-of-care (POC) testing for infections such as influenza A/B, COVID-19, respiratory syncytial virus (RSV), and Group A Streptococcus was widely introduced in primary care in recent years. This study investigates the patterns of oral antibiotic prescription in Poland during the pandemic and post-pandemic periods. **Methods**: We analyzed Polish National Healthcare Fund data on reimbursed oral antibiotics—Anatomical Therapeutic Chemical (ATC) J01 class—sold between 2019 and 2024. We quantified antibiotic groups by the number of packages sold and individual agents using the defined daily dose per 1000 inhabitants per day (DDD/TID). **Results**: Total oral antibiotic reimbursements noted a significant fall from 2019 to 2020 (20.9 million vs. 14.5 million packages reimbursed) and subsequently surged from 16.3 million packages in 2021 to 20.9 million in 2024. The most prescribed groups were penicillins (J01C), macrolides (J01F), and other beta-lactams (J01D). Amoxicillin with clavulanic acid was the most commonly used individual antibiotic, with its DDD/TID rising from an average of 2.3 to 2.6 in 2024. Although the use of phenoxymethylpenicillin initially decreased after the introduction of “strep-tests” in 2022 (DID 0.18 in 2023 vs. 0.23 in 2022), it increased again to 0.26 in 2024. **Conclusions**: Our findings demonstrate a slight continuous increase in oral antibiotic use in Poland, despite the current widespread availability of POC testing. The persistent and growing preference for amoxicillin with clavulanic acid, an agent not typically recommended as first-line treatment for most infections, suggests that antibiotic stewardship efforts need to continue in order to curb inappropriate prescription.

## 1. Introduction

Global antibiotic consumption rates are increasing and are estimated to continue to increase in the upcoming years. In 2023, the estimated total global consumption of antibiotics was 17.0 defined daily doses per 1000 inhabitants per day (DDD/TID) [1]. A temporary decrease was noted during the COVID-19 pandemic, especially in studies analyzing community-based data [2,3,4]; however, a subsequent rebound has been observed post-pandemic [1]. Antibiotic consumption is also increasing in Poland, and according to the Global Antimicrobial Resistance and Use Surveillance System (GLASS) report, in 2022, consumption of antibacterials in Poland reached 24.36 DDD/TID and was the sixth highest recorded consumption in the European region [5], which shows an increase in comparison with previous studies (22.2 DDD/TID in 2007 and 23.9 DDD/TID in 2016) [6].

Antibiotic consumption rates, along with inappropriate antibiotic use, are strongly correlated with antibiotic resistance [7], which is currently one of the leading health issues in the world. The global number of hospital-associated drug-resistant infections is estimated to be 136 million per year [8]. Apart from increasing healthcare costs due to prolonged hospitalizations, drug-resistant infections are also a significant cause of death, with an estimated 1.27 million deaths in the world attributed to bacterial resistance in 2019 [9].

In light of the elevated emergence of antibiotic-resistant bacteria strains and a low number of new antibiotics being invented, antibiotic stewardship becomes one of the most important challenges for public health. Multiple action plans, educational projects, and stewardship policy programs are being introduced globally. The World Health Organization (WHO) has developed the AWaRe Classification of antibiotics, which divides antibiotics into three groups with different recommendations for their use. The Access group includes antibacterials with a relatively low potential of resistance, which are recommended as the first or second choice for treatment. The Watch group includes high-priority antibiotics with higher resistance potential, which are the main targets of stewardship programs, being the first or second choice of treatment for specific diseases. The Reserve group includes antibiotics that should only be used in the treatment of confirmed multidrug-resistant infections [10,11]. The United Nations General Assembly has recommended that by the year 2030, substances from the Access group should constitute 70% of all antibiotics used globally [12].

Among the antibiotic stewardship efforts, point-of-care diagnostic tests were introduced to primary care in Poland to be available free of charge for patients. From 1 July 2022, Group A Streptococcus antigen tests (“strep-tests”) and quick CRP tests were introduced as reimbursed diagnostic methods in primary care. During the COVID-19 pandemic, quick antigen tests for SARS-CoV-2 and influenza were available in most clinics, and in January 2023, the full reimbursement of the three-in-one SARS-CoV-2, influenza, and RSV test was introduced. After introducing the full reimbursement, the point-of-care tests listed above have become available to be performed at the clinics, free of charge, at the doctor’s discretion to use them. All tests can be performed on any publicly insured patient, with the exception of quick CRP tests, which are only available for pediatric patients under 6 years of age.

Currently, it is estimated that up to 30–50% of antimicrobials are prescribed inadequately in hospital settings [13], and between 44% and 98% respiratory tract infections are treated inappropriately with antibiotics [14]. In total, 80 to 90% of all antibiotic prescriptions are issued by general practitioners [14]; hence, primary care is an important area for future antibiotic stewardship programs.

We aimed to analyze data on community antibiotic prescriptions in Poland in recent years based on the National Healthcare Fund reimbursement data, in order to assess the effectiveness of the current antibiotic stewardship programs, as well as to analyze current prescription patterns after the COVID-19 pandemic and the introduction of POC testing.

## 2. Results

**Figure 1:**
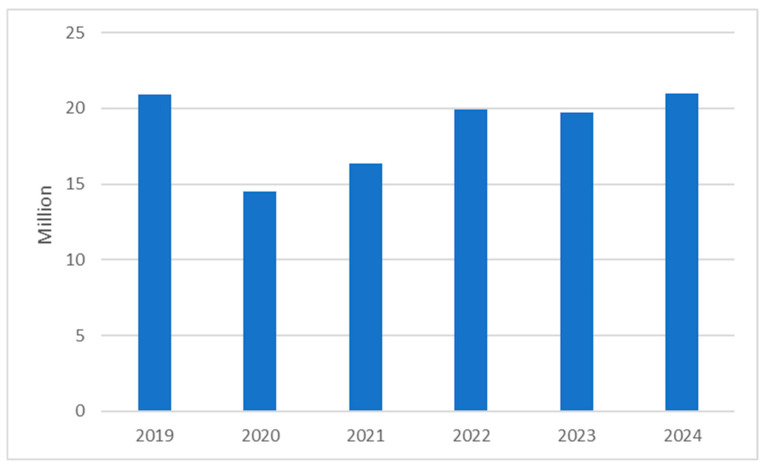
Total amount of packages of antibiotics reimbursed by the National Healthcare Fund in Poland in the years 2019–2024.

A decrease in the total use of oral antimicrobials in Poland was noted during the highest level of COVID-19 pandemic restrictions (from 20,881,550 packages of antibiotic medication reimbursed in 2019 to 14,515,213 in 2020 and 16,335,215 in 2021), with a subsequent gradual growth back up to pre-pandemic levels (20,971,156 packages reimbursed in 2024) (Figure 1).

**Figure 2:**
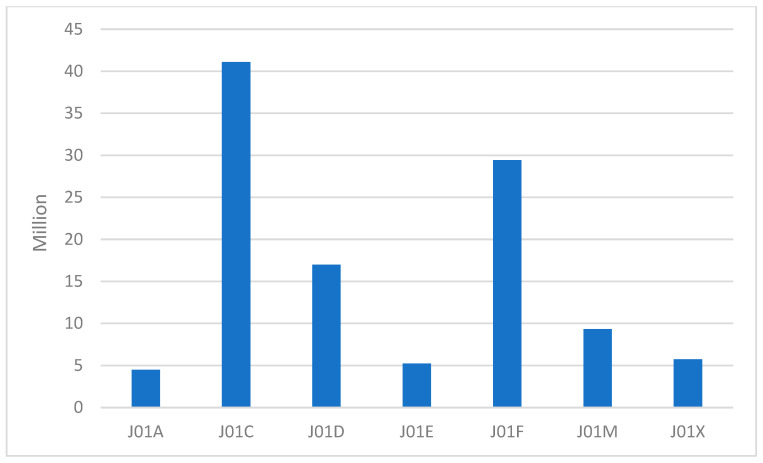
Total amount of packages reimbursed by the National Healthcare Fund in Poland in the years 2019–2024 by ATC group.

**Figure 3:**
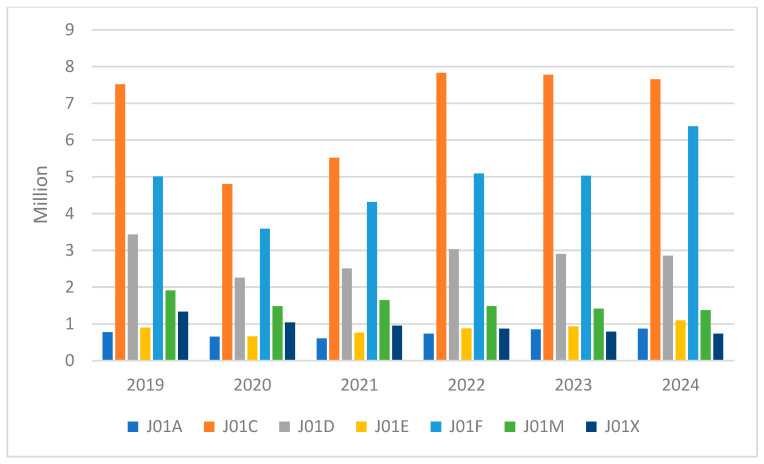
Yearly amount of packages reimbursed by the National Healthcare Fund in Poland in the years 2019–2024 by ATC group.

Over the last six years, the most commonly prescribed antibiotic groups in Poland have been consistently penicillins (J01C ATC group) with 41,099,162 packages sold in the years 2019–2024. Apart from the previously mentioned decline in the years 2020–2021, the level of utilization of this group has been stable, with only a slight increase between 2019 and 2024. The second most commonly reimbursed group comprised macrolides, lincosamides, and streptogramins (J01F ATC group, in which macrolides are the group with the most substances with reimbursement and suitable for oral administration), with 29,413,319 packages reimbursed in the years 2019–2024. The third group was other beta-lactams (J01D ATC group) with 16,992,072 packages reimbursed between 2019 and 2024 (Figure 2). The J01F (macrolides, lincosamides, and streptogramins) and J01E (sulfonamides and trimethoprim) ATC groups showed an increase in consumption since 2019, while the rates of consumption in J01D (other beta-lactam antibacterials), J01M (quinolone antibacterials), and J01X (other antibacterials) groups have decreased. The remaining ATC groups remained stable over the years, excluding the temporary decrease in 2020–2021, which involved all ATC antibiotic groups (Figure 3). Data on J01B and J01G groups were not represented, since there were no reimbursed oral antibacterials from those groups under the Polish National Healthcare Fund system.

**Figure 4:**
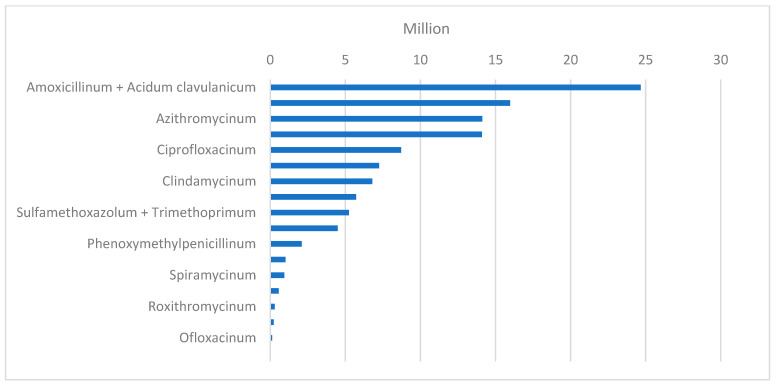
Total reimbursed antibiotics in the years 2019–2024 by the number of packages by substance.

In the analysis of single antibiotic prescription patterns, the defined daily dose per 1000 inhabitants per day (DDD/TID) was primarily used as it is a more reliable indicator than the amount of packages reimbursed. According to this indicator, the most commonly reimbursed antibiotic across all the years analyzed was amoxicillin with clavulanic acid, with a DDD/TID average of 2.31 and 24,646,339 packages sold in total in the past six years. It has accounted for 22% of all antibiotic reimbursements across the years analyzed. Apart from the 2020–2021 decline, the DDD/TID of amoxicillin with clavulanic acid has been steadily growing, and in 2024, this substance reached a DDD/TID of 2.61. The second most commonly prescribed antibiotic in the years 2021–2024 was cefuroxime with an average DDD/TID of 2.11. It constituted 14.2% of all antibiotic reimbursements in 2019–2024. Cefuroxime reached its highest prescription levels in 2019, with a DDD/TID of 2.38 and 3,165,504 reimbursed packages. Since then, the amount of prescriptions of cefuroxime has decreased slightly, and in 2024, it declined to 2,705,625 packages and a DDD/TID of 2.16. The third most commonly prescribed substance, by DDD/TID, was amoxicillin with an average DDD/TID value of 1.63. It constituted 12.5% of all antibiotic reimbursements across the years analyzed. Detailed data on DDD/TID and the number of packages sold yearly are shown in Table 1 and Figure 4.

**Figure 5:**
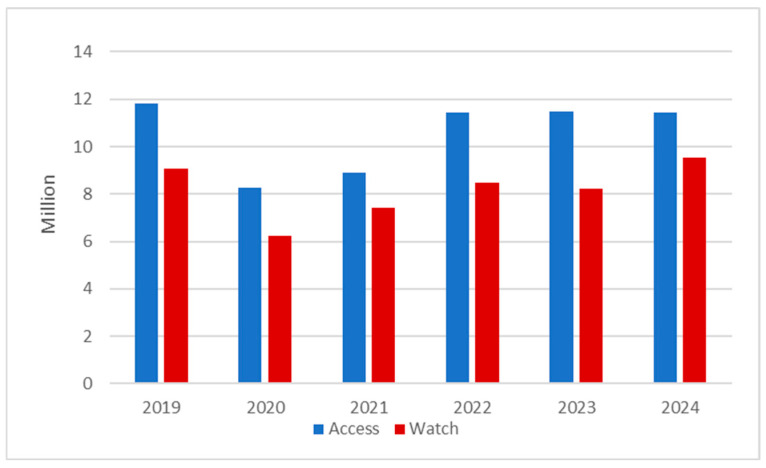
Yearly amount of packages reimbursed by the National Healthcare Fund in Poland in the years 2019–2024 by the WHO AWaRe categories.

According to the WHO AWaRe 2021 classification, which is used for the evaluation and monitoring of antibiotic use, substances are grouped into Access, Watch, and Reserve classes. Among the analyzed substances, none of the reimbursed antibiotics available for oral administration was grouped into the Reserve class, as this class generally includes antibiotics for parenteral, hospital-associated use in multidrug-resistant infections. Both Access and Watch classes of antibiotics showed a decrease in consumption during the COVID-19 period. In all the years analyzed, the use of antibiotics within the Access class was consistently higher than within the Watch class. However, while the Access class antibiotics reimbursements were stable and even decreased slightly between 2019 and 2024 (11,824,558 vs. 11,442,187 packages reimbursed), the Watch class consumption increased in the years analyzed (9,056,992 vs. 9,514,296 packages reimbursed). The highest increase in consumption of Watch class antibiotics was noted between the years 2023 and 2024 (8,212,332 vs. 9,514,296 packages reimbursed) (Figure 5). In all the years analyzed, the utilization of substances in the Access group did not reach the United Nations General Assembly goal of 70% of prescribed antibiotics. The percentage of the Access group utilization in 2019–2024 in total was 56.4%. In 2024, it reached the lowest value (54.6%) across all the years analyzed (Table 2).

**Figure 6:**
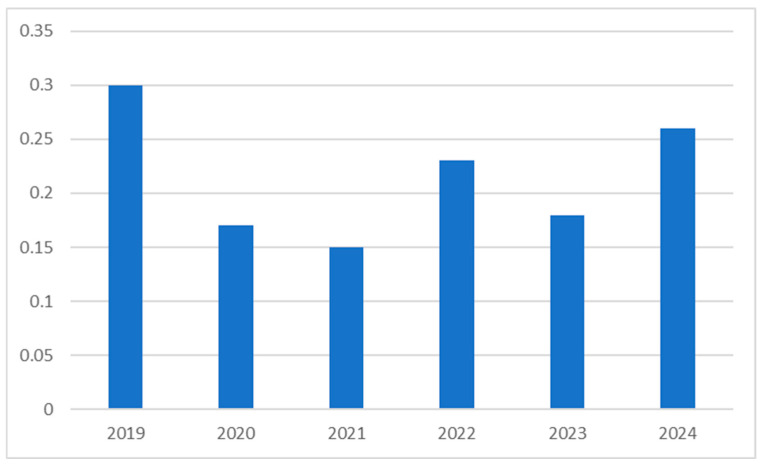
DDD/TID index of phenoxymethylpenicillin in the years 2019–2024.

Changes in phenoxymethylpenicillin use over time are an interesting focus point in the presented data. On 1 July 2022, quick antigen-detection tests for group A streptococcus (“strep-tests”) were introduced as a standard diagnostic modality in primary care, and thus, they became easily accessible to all family doctors in Poland. Interestingly, in 2023, the first full year after the introduction of the “strep-test” to primary care, the use of phenoxymethylpenicillin decreased (DDD/TID 0.18, compared to 0.23 in 2022), and in 2024, it increased again (DDD/TID 0.26) (Figure 6).

It is important to note that between October 2022 and December 2023, phenoxymethylpenicillin was included on periodical lists of medical products at risk of shortage in Poland [15], which might have contributed to this temporary reduction in use.

## 3. Discussion

The results of our study indicate a generally stable level of antibiotic consumption in Poland in the years 2019–2024, with a slight tendency to increase. The exceptions were the years 2020 and 2021, which aligned with the highest level of COVID-19 pandemic restrictions. Such reductions were previously noted in other studies analyzing antibiotic use in the pre- and post-pandemic periods [1,4], although the number of COVID-19 cases has also been positively associated with the consumption of certain antibiotic groups [4]. A pattern of rapid decrease during the COVID-19 pandemic and a gradual post-pandemic increase, as seen in our data, was noted in high-income countries in other studies [1]. In Poland, during the COVID-19 pandemic, the restrictions included obligatory mask-wearing in public areas, the closure of educational facilities, limiting the number of people present at a time in public places, and obligatory quarantine for patients with a confirmed COVID-19 infection. The most likely cause of the temporary decrease in antibiotic use was these restrictions, which reduced the incidence of influenza-like illnesses and other respiratory tract infections [16]. Respiratory tract infections are one of the main reasons for antibiotic prescription in primary care [17,18,19]; therefore, their lower rate of incidence results in a lower total antibiotic consumption.

In general, the J01 class (antibacterials in systemic use) can be considered to be used quite broadly among Polish general practice physicians, since respiratory tract infections and other infections are a common cause of their consultations. Although further studies on physician decision-making processes are necessary, it is reasonable to assume that part of the antibiotic use occurs contrary to guideline recommendations, most likely due to the overestimation of the likelihood of bacterial infection.

Among the ATC groups, the J01F group—macrolides, lincosamides, and streptogramins—has noted the biggest increase in prescriptions in the years analyzed. This should be attributed to the increase in oral macrolides consumption, given that within the J01F group, macrolides accounted for four out of five of the reimbursed orally administered substances. The only reimbursed oral lincosamide in Poland, clindamycin, did not have any significant increase in the number of prescriptions in recent years, and there were no reimbursed oral streptogramins in the Polish healthcare system in the years analyzed.

In recent years, an increase in the number of cases of pertussis has been noted in many European countries [20]. It is presumed that the pertussis resurgence is associated partly with a decreased vaccination coverage during the COVID-19 pandemic. Increased pertussis incidence has also been noted in Poland, where, based on data from the National Institute of Public Health, the number of reported pertussis cases in 2024 was around 35 times higher than in 2023 [21]. An increased number of pertussis cases was also likely to influence the number of macrolide prescriptions in non-appropriate situations, based on presuming a higher probability of pertussis infection in patients presenting with a cough without confirming the diagnosis with the appropriate tests.

Other factors contributing to macrolide overprescription include their broader coverage of atypical bacteria, which physicians may prefer for empiric treatment when diagnostic tests are unavailable in primary care. Furthermore, macrolides are an alternative for patients reporting intolerance or sensitivity to beta-lactam antibiotics. Finally, the simplicity of the dosage regimen—such as a 3-day, once-daily course for azithromycin—may appeal to both doctors and patients, potentially influencing prescription choice. A key disadvantage, however, is the more frequent clinical failure risk associated with macrolides due to the high rate of Streptococcus pneumoniae resistance relative to amoxicillin [22]

Additionally, COVID-19 cases have been positively correlated with the level of macrolide consumption [4,23]. On the contrary, a study conducted in Northern Ireland noted a decrease in macrolide prescriptions during the COVID-19 pandemic [24]. According to our data, a slight increase in the consumption of macrolides in comparison with other antibiotic groups was present during the COVID-19 pandemic; however, the biggest increase in macrolide reimbursements was noted in the post-pandemic period.

According to our study, the three most commonly reimbursed antibiotic groups in Poland were penicillins, macrolides, and other beta-lactams. Similar results have been noted in other countries. Two studies conducted in Northern Ireland and England found penicillins, tetracyclines, and macrolides to be among the three most commonly prescribed groups [24,25].

While the antibiotic groups that are utilized by the physicians in Poland do not differ from the standard use in different European countries, the most commonly used substances clearly differ from those primarily selected in different countries.

In the years analyzed, the most commonly reimbursed antibiotic in Poland was amoxicillin with clavulanic acid. It is a worrying observation, which might suggest that clinicians in Poland have a tendency to choose a broader-spectrum beta-lactam as the empiric treatment instead of a moderate-spectrum, first-line choice of treatment, such as amoxicillin. While amoxicillin with clavulanic acid has been observed as one of the most commonly prescribed antibiotics in outpatient settings in previous studies [26,27], it has rarely been reported as the most commonly first-prescribed antibiotic, which might suggest a local problem in antibiotic prescription management.

According to a recent study published in 2025, amoxicillin (including amoxicillin in combination with clavulanic acid) was the most commonly prescribed antibiotic in France, Belgium, Germany, Italy, the UK, and Poland. The study did not make a distinction between amoxicillin with and without clavulanic acid [28]. In a previous study conducted in non-EU southern and eastern states, it was found that amoxicillin with clavulanic acid was most commonly used in Turkey and Georgia (13 DID and 9 DID, respectively) [29]. A study based on general practice clinician surveys reported that Poland, along with Greece, Georgia, Moldova, Croatia, and Romania, showed a tendency to use amoxicillin with clavulanic acid or macrolides in a significant portion of respiratory tract infection consultations. The study found that Polish practitioners prescribed amoxicillin with clavulanic acid in 19.2% of consultations, while, in comparison, in Denmark it was only used in 9.7% of consultations. Other countries with low amoxicillin with clavulanic acid consumption were Germany, Ireland, and the United Kingdom [30]. In another study conducted in England, amoxicillin was the most commonly prescribed antibiotic, while penicillin combinations (99% of which were amoxicillin with clavulanic acid) constituted only around 10% of penicillin prescriptions [25]. The tendency to use broad-spectrum penicillins among Polish physicians might be a result of an underestimation of the effectiveness of first-choice, narrow-spectrum antibiotics. It might also have been influenced by patients’ inquiries, who are often concerned about receiving a “less effective” treatment. Further studies on doctor and patient behaviors and decision-making processes are necessary to confirm the reasons for the overuse of broad-spectrum antibiotics.

Based on the comparisons of antibiotic use in different countries, as mentioned above, it can be observed that while Poland is currently considered a high-income country, the antibiotic prescriptions, at least partly, still follow the middle-income country patterns, such as Turkey and Georgia.

The WHO has published the AWaRe antibiotics classification to educate and aid clinicians in decisions on antibiotic usage [11]. It has also been used in antibiotic consumption reports to help assess the level of inappropriate antibiotic administration [12]. In our study, we noted an underutilization of the Access group of antibiotics, with the consequential overutilization of the Watch group. In none of the years analyzed did the consumption of reimbursed antibiotics in Poland reach the goal of 70% Access group of substances. Difficulties in reaching this goal have been reported in previous studies using the AWaRe classification to assess the antibiotic prescription patterns [12,31,32]. Failure to achieve the WHO-recommended target of 70% Access-group antibiotic consumption in Poland is largely attributable to the high volume of prescriptions for macrolides and cefuroxime, which are designated as Watch group agents.

The widespread use of cefuroxime in Poland remains poorly understood, as no clinical evidence clearly justifies its preference over amoxicillin. Cefuroxime does not cover atypical bacteria and demonstrates lower efficacy against Streptococcus pneumoniae compared with amoxicillin. This preference contradicts Polish national guidelines, which do not list cefuroxime as a first-line therapy for community-acquired respiratory tract infections. One of the reasons for cefuroxime overuse might be the overestimation of amoxicillin allergies. In cases of non-immediate reactions to amoxicillin, Polish guidelines recommend using cefuroxime for many infections, such as sinusitis, otitis media, and community-acquired pneumonia in children, which might lead physicians to prescribe it if sensitivity is suspected, even if amoxicillin allergy is not clearly confirmed. Further studies are necessary to verify this association.

An interesting observation from an antibiotic stewardship point of view is the level of phenoxymethylpenicillin consumption in recent years in Poland. In group A Streptococci tonsillitis, phenoxymethylpenicillin was the first-line treatment, given the lack of observed resistance of group A Streptococci to penicillin so far [33]. With the introduction of quick antigen-detection tests for group A streptococcus to primary care clinics in Poland, a significant increase in consumption of phenoxymethylpenicillin might have been expected. However, after a temporary decrease in the year following the introduction of “strep-tests”, most likely caused by pharmacy shortages, only a slight increase in phenoxymethylpenicillin consumption was noted in 2024. While the shortages may have temporarily influenced phenoxymethylpenicillin consumption levels, it should be investigated whether this continued low-level effect of this new diagnostic modality is due to its low utilization by primary care physicians, or to the possibility that correctly diagnosed group A Streptococci tonsillitis is still being treated inappropriately by clinicians in Poland.

### Limitations

Certain limitations of the study need to be taken into account. The National Healthcare Fund only provided data on the prescriptions of reimbursed medical products. Antibiotics prescribed at full price, without reimbursement from the National Health Fund, were not included in this study. The non-reimbursed substances are generally less commonly used, which minimizes the influence of this limitation on the presented data.

Additionally, the analyzed data only included information on the amount of prescription reimbursements. Information on the clinical situation and outcomes, reasons for prescription, and diagnostic tests performed prior to treatment, including point-of-care tests, was not analyzed. Due to this limitation, a definite correlation between prescription patterns and factors, such as public health conditions, point-of-care tests, or antibiotic stewardship efforts, cannot be established based on the presented data.

The analyzed data did not undergo a time-series analysis to test the statistical significance of the influence of outside factors, such as the introduction of point-of-care tests, on the patterns of antibiotic reimbursements.

## 4. Materials and Methods

Data on antibiotic consumption were extracted from publicly available information published by the Polish National Healthcare Fund (Narodowy Fundusz Zdrowia). Data on the number of packages sold with a reimbursement from the healthcare system in each year analyzed were retrieved. DDD/TID was calculated for each substance from the J01 class in the ATC classification for which reimbursement data was available. For the DDD/TID calculation, defined daily doses (DDD) were used from the ATC/DDD Index 2025 published by the Norwegian Institute of Public Health in collaboration with the World Health Organization.

The data on the number of packages of each substance and groups of antibiotics and DDD/TID of each substance were analyzed and compared on a year-to-year basis.

Data on antibiotics registered for parenteral routes of administration were excluded. The study’s main goal was to gather information representative of antibiotic consumption in the outpatient healthcare setting. Antimicrobials administered via non-oral routes are mostly used in hospital settings, and therefore their consumption is influenced by different factors than oral antimicrobial consumption. Furthermore, the reimbursement data might not be representative of the infections treated in the hospital settings.

## 5. Conclusions

Apart from the temporary decrease during the COVID-19 pandemic restrictions, the sales of antibiotics in recent years have been steady, with a slight tendency toward growth. COVID-19 restrictions were the likely cause of the reduction in antibiotic reimbursements in 2020–2021; however, they did not have a lasting influence on total antibiotic sales in Poland.

Based on the presented data, we can conclude that the clinicians in Poland have a tendency to overuse broad-spectrum antibiotics, including substances from the Watch group of the WHO AWaRe classification. Instead of moving towards the objective of 70% Access group antibiotic prescriptions, the sales in Poland have been gradually moving away from that level. The persistent and growing preference for amoxicillin with clavulanic acid, an agent not typically recommended as first-line treatment for most infections, suggests that further antibiotic stewardship projects must be introduced in order to improve antibiotic prescription patterns and delay the growth of antibiotic resistance.

## Figures and Tables

**Table 1:** DDD/TID and the total number of packages of analyzed antibiotics reimbursed by the National Healthcare Fund in the years 2019–2024.

Substance	Data	2019	2020	2021	2022	2023	2024
Amoxicillinum	DDD/TID	1.8	1.1	1.2	1.9	2.0	1.8
	Packages	2,572,174.7	1,531,603.5	1,784,038.0	2,770,052.0	2,865,151.9	2,576,892.6
Amoxicillinum + Acidum clavulanicum	DDD/TID	2.4	1.7	2.0	2.6	2.6	2.6
	Packages	4,429,831.7	3,026,796.2	3,471,137.3	4,615,150.3	4,573,729.2	4,556,501.6
Azithromycinum	DDD/TID	0.7	0.5	0.7	0.9	0.9	1.1
	Packages	2,065,730.3	1,485,001.5	2,123,616.6	2,704,242.5	2,564,377.9	3,181,548.0
Cefaclorum	DDD/TID	0.1	0.1	0.1	0.1	0.0	0.1
	Packages	263,861.3	128,563.9	173,271.1	193,313.3	107,404.2	147,791.4
Cefadroxilum	DDD/TID	0.0	0.0	0.0			
	Packages	3247.2	3764.8	3330.0			
Cefuroximum	DDD/TID	2.4	1.7	1.8	2.3	2.3	2.2
	Packages	3,165,504.5	2,127,582.1	2,330,548.2	2,839,890.3	2,798,374.5	2,705,624.8
Ciprofloxacinum	DDD/TID	0.6	0.5	0.5	0.5	0.5	0.5
	Packages	1,666,343.8	1,325,133.8	1,459,006.1	1,482,424.7	1,412,655.1	1,376,163.8
Clarithromycinum	DDD/TID	1.4	0.7	0.8	0.9	1.0	1.6
	Packages	1,301,564.7	787,577.0	918,058.5	1,113,067.2	1,185,297.8	1,947,671.6
Clindamycinum	DDD/TID	0.5	0.4	0.4	0.5	0.5	0.5
	Packages	1,292,284.7	1,089,843.4	1,066,114.9	1,124,298.4	1,138,262.1	1,091,458.2
Cloxacillinum	DDD/TID			0.0	0.0	0.0	0.0
	Packages			42,924.1	54,922.2	60,111.4	73,778.7
Doxycyclinum	DDD/TID	0.6	0.5	0.5	0.5	0.6	0.7
	Packages	776,139.1	655,859.8	606,266.4	734,172.3	850,919.9	871,509.5
Furazidinum	DDD/TID	0.6	0.5	0.4	0.4	0.4	0.4
	Packages	1,336,026.0	1,039,743.9	951,708.0	872,134.0	787,758.0	733,768.0
Norfloxacinum	DDD/TID	0.2	0.1	0.1			
	Packages	217,202.6	158,055.9	190,464.7			
Ofloxacinum	DDD/TID	0.0	0.0	0.0	0.0	0.0	0.0
	Packages	26,566.4	20,254.0	19,836.0	19,651.0	15,287.0	14,673.0
Phenoxymethylpenicillinum	DDD/TID	0.3	0.2	0.2	0.2	0.2	0.3
	Packages	513,323.7	247,575.6	221,651.3	389,796.6	277,508.5	444,510.8
Roxithromycinum	DDD/TID	0.0	0.0	0.0	0.0	0.0	0.0
	Packages	104,481.9	54,350.3	59,668.8	25,597.0	22,619.8	27,097.0
Spiramycinum	DDD/TID	0.1	0.0	0.0	0.0	0.0	0.0
	Packages	245,736.4	169,792.2	149,048.5	124,910.1	121,602.3	128,399.4
Sulfamethoxazolum + Trimethoprimum	DDD/TID	0.2	0.2	0.2	0.2	0.2	0.3
	Packages	901,531.0	663,714.7	764,526.7	879,765.4	931,130.8	1,093,767.9

**Table 2:** Yearly amount of packages reimbursed by the National Healthcare Fund in Poland in the years 2019–2024 by the WHO AWaRe categories and the ratio of Access category reimbursements.

Year	Access	Watch	Total	% of Access Category Reimbursements
2019	11,824,558.1	9,056,991.8	20,881,549.9	56.63%
2020	8,258,901.93	6,236,056.7	14,494,958.6	56.98%
2021	8,911,696.63	7,403,682.5	16,315,379.1	54.62%
2022	11,440,291.2	8,483,445.1	19,923,736.3	57.42%
2023	11,484,571.8	8,212,331.6	19,696,903.4	58.31%
2024	11,442,187.3	95,14,295.9	20,956,483.2	54.60%
Total	63,362,206.8	48,906,804	112,269,010	56.44%

## Data Availability

Data analyzed in the study were derived from the following resources available in the public domain: National Healthcare Fund–Statistics (*Narodowy Fundusz Zdrowia—Statystyki*), available at: https://statystyki.nfz.gov.pl/ (accessed on: 20 July 2025).

## Abbreviations

The following abbreviations are used in this manuscript:

POC	Point of care
RSV	Respiratory syncytial virus
ATC	Anatomical therapeutic chemical
DDD/TID	Defined daily dose per 1000 inhabitants per day
GLASS	Global antimicrobial resistance and use surveillance system
WHO	World Health Organization
DDD	Defined daily dose
